# Robotic-assisted cholecystectomy is superior to laparoscopic cholecystectomy in the initial training for surgical novices in an ex vivo porcine model: a randomized crossover study

**DOI:** 10.1007/s00464-021-08373-6

**Published:** 2021-02-26

**Authors:** E. Willuth, S. F. Hardon, F. Lang, C. M. Haney, E. A. Felinska, K. F. Kowalewski, B. P. Müller-Stich, T. Horeman, F. Nickel

**Affiliations:** 1 Department of General, Visceral and Transplantation Surgery, Heidelberg University Hospital, Heidelberg, Germany; 2 Department of Surgery, Amsterdam UMC—VU University Medical Center, Amsterdam, The Netherlands; 3 Department of Urology and Urological Surgery, University Medical Center Mannheim, Heidelberg University, Mannheim, Germany; 4 Department of BioMechanical Engineering, Delft University of Technology, Delft, The Netherlands

**Keywords:** Laparoscopy, Robotic surgery, Education, Cholecystectomy-randomized controlled trial

## Abstract

**Background:**

Robotic-assisted surgery (RAS) potentially reduces workload and shortens the surgical learning curve compared to conventional laparoscopy (CL). The present study aimed to compare robotic-assisted cholecystectomy (RAC) to laparoscopic cholecystectomy (LC) in the initial learning phase for novices.

**Methods:**

In a randomized crossover study, medical students (*n* = 40) in their clinical years performed both LC and RAC on a cadaveric porcine model. After standardized instructions and basic skill training, group 1 started with RAC and then performed LC, while group 2 started with LC and then performed RAC. The primary endpoint was surgical performance measured with Objective Structured Assessment of Technical Skills (OSATS) score, secondary endpoints included operating time, complications (liver damage, gallbladder perforations, vessel damage), force applied to tissue, and subjective workload assessment.

**Results:**

Surgical performance was better for RAC than for LC for total OSATS (RAC = 77.4 ± 7.9 vs. LC = 73.8 ± 9.4; *p* = 0.025, global OSATS (RAC = 27.2 ± 1.0 vs. LC = 26.5 ± 1.6; *p* = 0.012, and task specific OSATS score (RAC = 50.5 ± 7.5 vs. LC = 47.1 ± 8.5; *p* = 0.037). There were less complications with RAC than with LC (10 (25.6%) vs. 26 (65.0%), *p* = 0.006) but no difference in operating times (RAC = 77.0 ± 15.3 vs. LC = 75.5 ± 15.3 min; *p* = 0.517). Force applied to tissue was similar. Students found RAC less physical demanding and less frustrating than LC.

**Conclusions:**

Novices performed their first cholecystectomies with better performance and less complications with RAS than with CL, while operating time showed no differences. Students perceived less subjective workload for RAS than for CL. Unlike our expectations, the lack of haptic feedback on the robotic system did not lead to higher force application during RAC than LC and did not increase tissue damage. These results show potential advantages for RAS over CL for surgical novices while performing their first RAC and LC using an ex vivo cadaveric porcine model.

**Registration number:**

researchregistry6029

**Graphic abstract:**

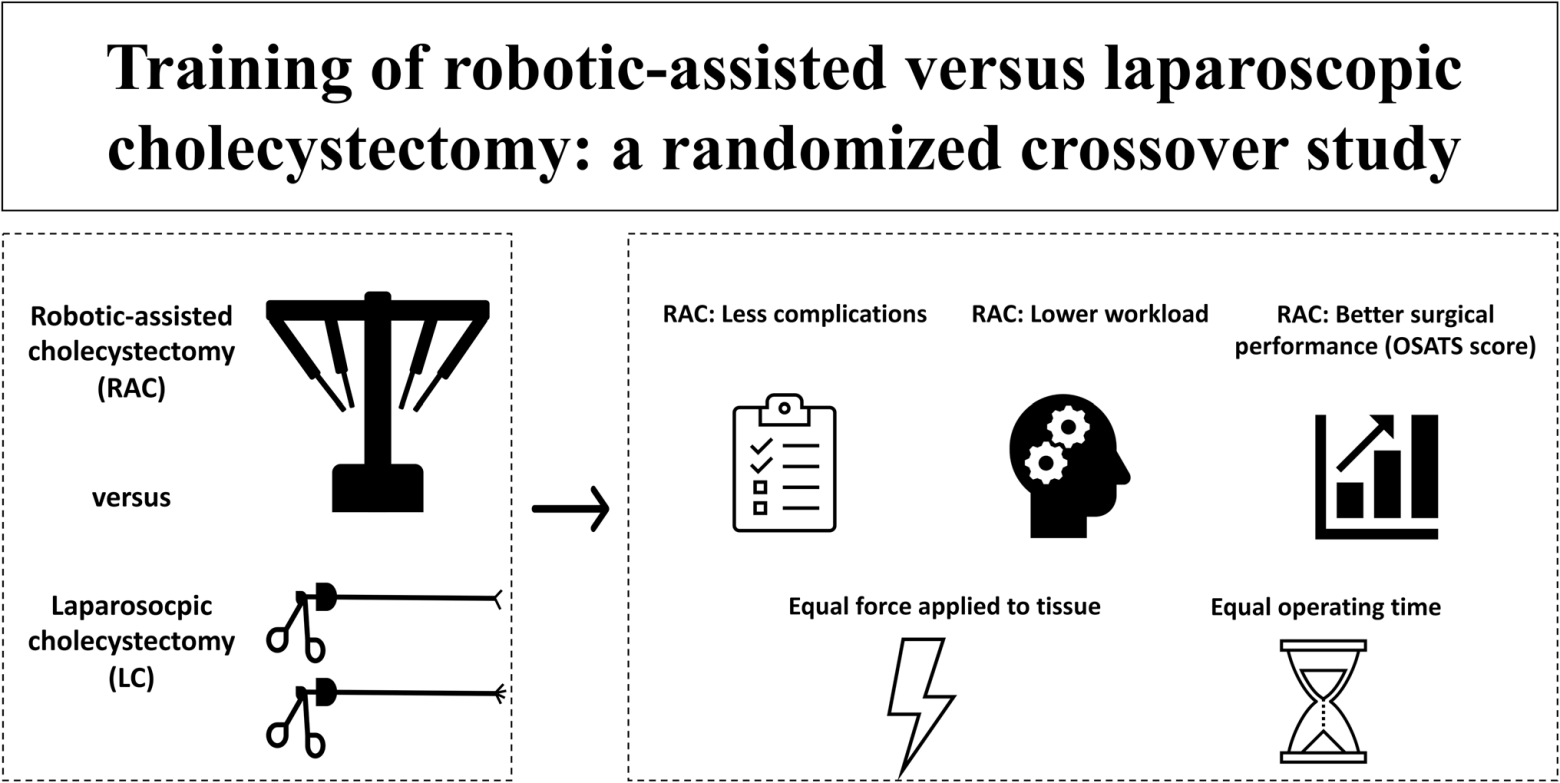

Robotic-assisted surgery (RAS) provides theoretical advantages compared to conventional laparoscopy (CL) such as a better ergonomics, additional degrees of freedom of the instruments, a more precise transfer of the operator’s action and a stable 3-dimensional view of the operating field [1, 2].These features have been developed to facilitate minimally invasive surgery (MIS) by compensating the downsides of CL such as pivot and fulcrum effect, difficult instrument handling and hand–eye coordination [3]. 3D vision in laparoscopy helps reducing operative time and complication rate, especially for laparoscopic suturing [4]. Both mental and physical workload can be reduced [2, 5] and especially for novices these features of RAS have the potential to shorten learning curves compared to CL [1, 6–10]. It has already been shown that RAS can improve the early learning curve for novices while learning an advanced laparoscopic task such as knot tying. On the other hand, the current robotic systems do not provide haptic feedback and therefore require surgeons to learn visual haptics during the learning curve to compensate for the lack of tissue feeling and to avoid tissue damage [11]. For novices, the missing haptics can be challenging while performing on the robot and may lead to higher tissue damage compared to CL [12]. A dedicated force measurement system for tracking, monitoring and assessment called ‘ForceSense’ (MediShield B.V., Delft, The Netherlands) is available for box trainers. It has been shown during surgical training that the monitoring of force, motion, and time parameters could help determine the participants skills level [13, 14]. Laparoscopic training with force feedback showed to be effective in determining development of basic laparoscopic tissue manipulation skills and helped improving surgical skills and self-confidence [15]. Haptic feedback showed to be beneficial for surgical novices in the early stages of their training as its presence seems to potentially help increase performance, safety, and training efficiency in MIS [16].

Even though RAS is considered as standard for several procedures, such as prostatectomy in urology, its use remains highly discussed in abdominal surgery and there is little evidence to support its clinical use. Most randomized clinical studies on the use of robotic systems showed operative outcomes were comparable between robotic-assisted and laparoscopic procedures but the cost was actually increased for robotic procedures [17, 18]. Overall surgical performance, complication rates and/or operative outcomes have not shown clear advantages with the robotic system in comparison to CL in randomized trials thus far but only in non-randomized studies [19–21]. Nevertheless, it has been shown that the level of prior laparoscopic experience affects the surgical outcome: surgeons with more advanced laparoscopic experience seemed to learn robotic tasks slower than laparoscopically inexperienced surgeons, as the experts had to “unlearn” laparoscopic techniques to be able to acquire robotic skills [22]. Incorporating robotics in laparoscopic training programs for medical students and surgical residents may shorten the clinical learning phase for RAS [23, 24].

This study aimed to compare RAS and CL in the initial learning phase for minimally invasive cholecystectomy in novices with regards operative performance, operating time, errors, excess force and subjective workload.

## Materials and methods

### Participants

Medical students in their clinical years of study with 10 h prior basic MIS training experience were invited to participate in this study. Participants with more experience in laparoscopic and robotic surgery were excluded. The participation was voluntary, and the participants were allowed to leave the study at any time. The participants received information about the study and informed consent was obtained. The local ethics committee at Heidelberg University approved the study protocol before inclusion of the trainees (S-436/2018). The cadaveric porcine livers used for the training of operations were obtained as side products from the local food industry.

### Setting and study design

The study was designed as a prospective monocentric randomized crossover study. The students were randomized in a 1:1 ratio in two groups and these were compared for the effectiveness of training a basic operation in CL and RAS. Each participant performed one laparoscopic cholecystectomy (LC) and one robotic-assisted cholecystectomy (RAC) on cadaveric porcine livers. In accordance with the crossover study design, participants from group 1 started with the RAC and performed the LC afterward; group 2 performed the LC first and then the RAC.

**Fig. 1:**
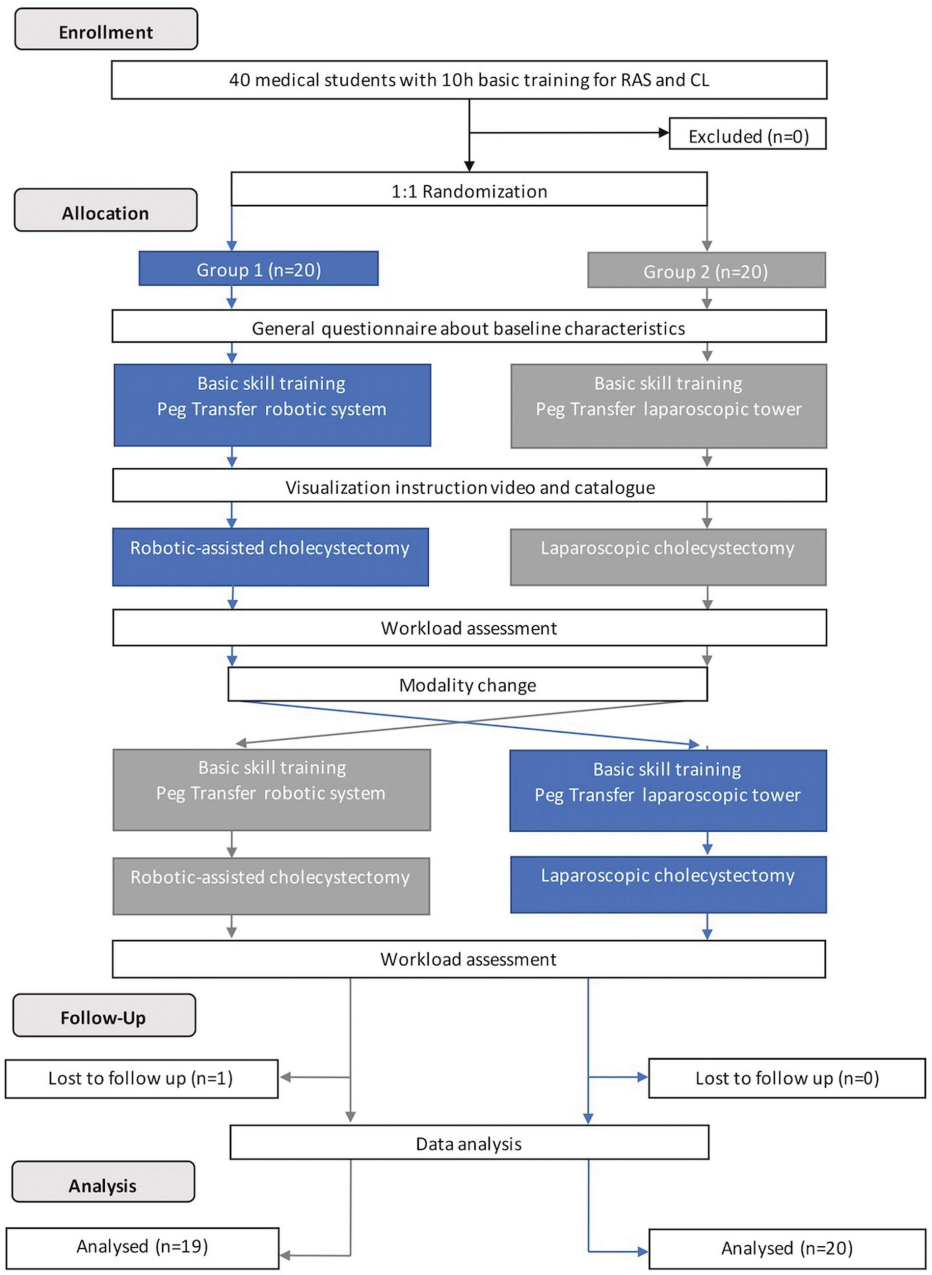
Flowchart

Before performing the cholecystectomies, all participants received standardized practical and theoretical instructions and performed a standardized basic skill training for RAS and CL. After completing each cholecystectomy, the students filled in post-procedure questionnaires. This study compared RAC and LC performed on porcine livers by novices as single assessments, therefore the present setting compared the first robotic and laparoscopic performances of this procedure in an ex vivo model, describing it as initial training performance. The study was designed, evaluated, and reported in line with the CONSORT criteria and was carried out in the training center and experimental operating room for MIS at the Department of General, Visceral, and Transplantation Surgery at Heidelberg University Hospital [25] (Fig. 1). The study was retrospectively registered via Research Registry (www.researchregistry.de, researchregistry6029).

### Practical and theoretical introduction for robotic-assisted surgery and conventional laparoscopy

All the participants received identical preparation to perform the cholecystectomies. For the standardized practical and theoretical instructions, students watched a teaching video and went through an instructional catalog. The recordings for the video were made previous to the study by the study director. The video and the catalog showed the students all the key steps necessary to perform the cholecystectomy in the box trainer and every step was explained showing an image from a previously recorded LC and RAC, side by side. The students watched it once, before performing their first cholecystectomy. To provide a standardized practical preparation, the students had to perform a basic skill training for RAS and CL using the Peg Transfer task. Students performed the Peg Transfer on the Da Vinci Surgical System (Intuitive Surgical, Sunnyvale, California, USA) before performing the RAC and performed it on the CL tower before the LC.

### Laparoscopic cholecystectomy

To perform the cholecystectomy, cadaveric porcine livers were used. The livers were secured in a force measurement device (further explanations below) and then placed in a Szabo-Berci-Sackier Box Trainer. Port placement and docking were not included in the procedures. Instruments were inserted via trocars and a standard 2D laparoscopic tower was used as this was hospital standard at the time of the study and still is in many hospitals. To perform the LC, the students used one fenestrated grasper and curved scissors (from KARL STORZ GmbH & Co. KG, Tuttlingen, Germany) along with clip applicators. Students had to dissect the cystic artery and the cystic duct to ensure a reliable identification of the structures. The cystic artery and the cystic duct had to be then clipped and cut. The gallbladder was then removed from the liver bed using the curved scissors. The trainees were instructed and evaluated by tutors during the procedure. The student’s performance was video recorded so that external evaluators could perform a blinded objective evaluation (only the screen was recorded). The total time of the operation was limited to 90 min, whereby the first part should be completed after 45 min in order to have enough time for the actual gallbladder removal.

### Robotic-assisted cholecystectomy

Setting and material was the same as for the LC, except for the instruments. To perform the RAC all students used curved scissors (8 mm) from Intuitive™ for Da Vinci *Si*. To hold the gallbladder students used either a fenestrated bipolar forceps (8 mm), a Maryland bipolar forceps (8 mm) or a Tenaculum forceps (8 mm) from Intuitive™. Instructions, evaluation and operating time measurement were identical to the LC.

### ForceSense tissue interaction assessment

**Fig. 2:**
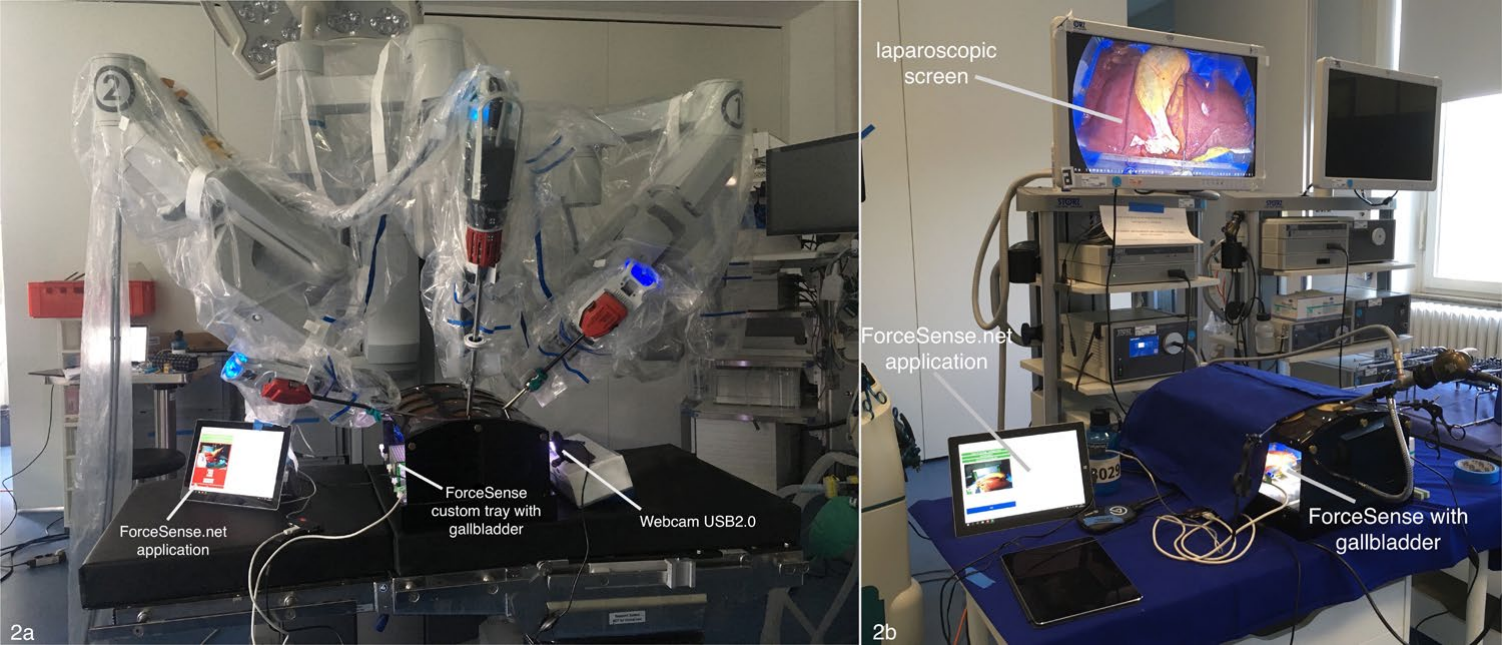
Force measurement setup for **A** Robotic-assisted surgery **B** Conventional laparoscopy

**Fig. 3:**
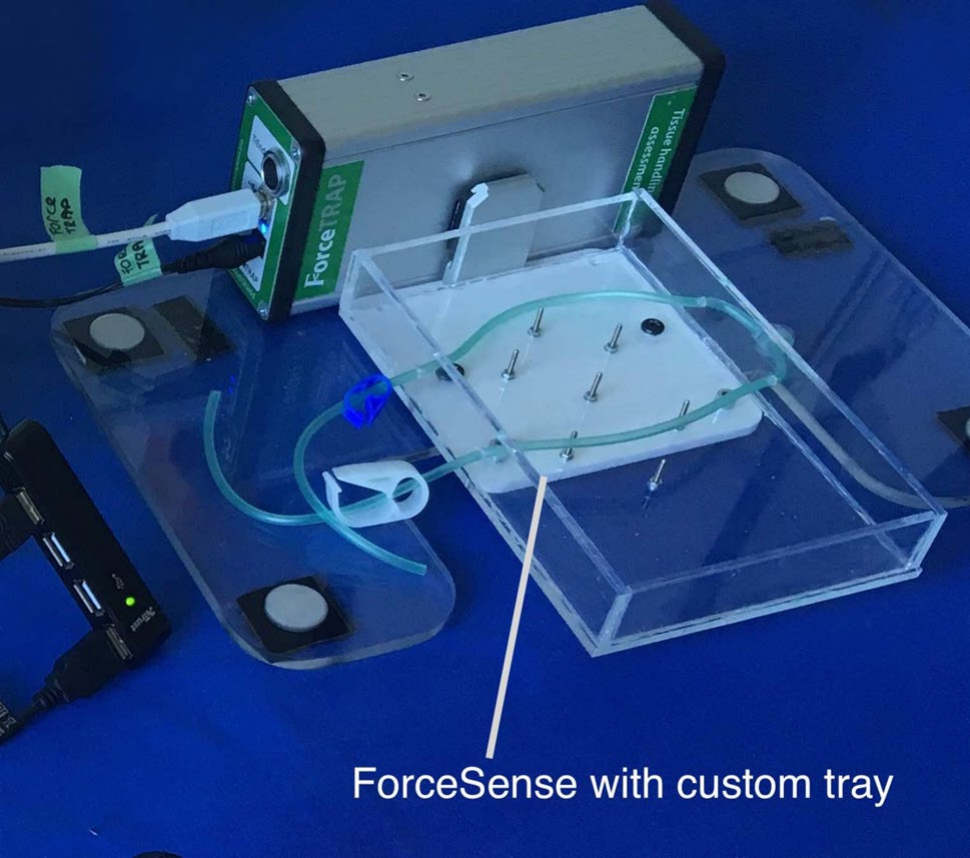
‘ForceSense’ application force measurement device with custom tray for mounting the liver with gallbladder

The setting for force measurements was the same on the Da Vinci System and on the laparoscopic tower. To ensure a standardized performance and to guarantee an optimal fixation of the liver, it had to be prepared and cut to a size of 10 cm in the width and 15 cm in the length. The force parameters used in this study are presented in Table 1 and are considered representative for delicate tissue manipulation- and instrument handling skills identification. To measure the interaction force, the system was linked to a custom basket that contained the prepared liver during the experiment (Fig. 2). Fixation pins were used to prevent relative displacement of the tissue in the basket parameters and to transfer all manipulation forces to the ForceSense force measurement table (Fig. 3). To calibrate compensation for the weight of the specimen, the participants were asked not to touch the training task or specimen with the instruments during the first 10 s of the measurement. This allowed the system to determine the mass of the specimen and to set a new force baseline by subtracting the FZ value from the following experimental force data represented in Fx, Fy and Fz. As the maximum measurement time for ForceSense is 10 min a new measurement was started every 10 min during the experiment till the task was completed. Afterward, the data was fused together with a separate algorithm by MediShield BV. When a recording was not completed, the measurement was excluded. For the maximum impulse, the first recording of every cholecystectomy was used, as all following measurements contained large moments with traction on the tissues for efficient dissection resulting in non-relevant outcomes. The ForceSense tissue interaction assessment was used for the first time in the context of a surgical procedure. This part of the study thus aimed to show that force measurements of this kind are able to identify and evaluate relevant force interaction events during complex laparoscopic tasks such as the laparoscopic cholecystectomy. The force measurement setup was introduced after start of the present study for exploratory purposes and evaluation of tissue interaction assessment on robotic-assisted and laparoscopic cholecystectomy with 18 participants.

**Table 1:** Force measurement parameters

Parameter	Description
Max absolute force	*The maximal force (Newton)* found in a trial indicating the largest jerk or punch in instrument–tissue interactions [12, 13]
Mean force during tissue manipulation	*I.e., Mean Force Non-Zero (Newton)* Indicating the averaged mean absolute force of periods during training the absolute force is not non-zero [12, 13]
Max impulse	I.e., Force Peak or Max Force Area [Ns]. When plotting force against time, Max Impulse is divined as the period with the highest absolute area under the force graph between the moment the force reached levels became higher than 0 till it reached zero again [12, 13]
Force Volume	Force volume (FV): Indicating the volume of an ellipsoid spanned around the standard deviations (SD) of the force along the three main principal components (PC’s). The largest SD found in the 3D force defines the orientation of PC1. The second largest SD defines the orientation of PC2 perpendicular to the first. PC3 oriented perpendicular to PC1 and PC2 [12, 13]

### Outcome measures

#### Primary endpoint

The participants performance during RAC versus LC was rated with a standardized and validated tool for assessing surgical skills: the total Objective Structured Assessment of Technical Skill score (OSATS) was used as primary endpoint [26, 27]. The global OSATS score evaluated tissue respect, efficiency, usage and knowledge of the instruments, camera assistance and workflow (35 reachable points). The task specific OSATS score assessed retraction of gall bladder and Calot triangle preparation, preparation of the cystic duct, cystic artery and gallbladder, clipping and cutting, knowledge of the procedure and the quality of the end product (70 reachable points).

#### Secondary endpoints

Secondary endpoints included operating time (in minutes) needed for the cholecystectomies and occurrence of complications in comparison between robotic-assisted and laparoscopic cholecystectomy. Complications were defined as liver damage, perforations of the gallbladder, damage to arteries and ducts, as well as misplacements of clips. The assessment of liver damage focused on the injuries caused on the gallbladder bed by the student during the removal, using a 3-point Likert scale. Further possible complications such as perforations, injuries on cystic artery and/or cystic duct and clipping were assessed using this same scale (Table 2). A combined endpoint was calculated to compare the occurrence of major damage on liver, gallbladder, vessels or through clips between RAC and the LC.

**Table 2:** Complication assessment for the cholecystectomies

	Rating scale	Definition
Liver damage		
None	0	No injuries on the gallbladder bed during removal
Minor damage	1	One small or superficial injury on the gallbladder bed
Major damage, many small lesions	2	One large or deep injury on the gallbladder bed; or many small or superficial lesions on the gallbladder bed
Perforations		
None	0	No gallbladder perforation
Minor damage	1	One small perforation, avoiding the spilling of gallbladder stones
Major damage	2	One large perforation without avoiding the spilling of gallbladder stones; or many small perforations
Damages on artery and duct		
None	0	No damage on artery and duct
Repairable damage	1	Artery and/or duct damaged, preparation and clipping still possible
Irreparable damage	2	Artery and/or duct sectioned
Clips placed		
All placed correctly	0	Artery and duct clipped correctly
Slightly out of correct place	1	Clips on artery and/or duct slightly out of place
Structure damaged, clips not closing	2	Artery and/or duct damaged, clips not closing on artery and/or duct

Finally, the student’s workload after performing each cholecystectomy was assessed using three questionnaires. The validated NASA Task Load Index (NASA TLX) assessed mental, physical and temporal demand such as frustration level and overall performance [28]. The After-Scenario-Questionnaire (ASQ) assessed how satisfied the students were with the ease to complete the task, the amount of time it took and the given informational support. This questionnaire allows a scenario-based assessment of participant’s satisfaction [29, 30]. Ultimately, a self-made questionnaire using a 5-Point Likert scale assessed physical workload and the ease to work on each operating system.

To assess the force applied to the tissue during the procedures the ForceSense tissue interaction assessment was used. The force interaction assessment was done as an exploratory pilot evaluation as this force measurement device was used for the first time in this setting.

#### Randomization

The study participants were randomized in a 1:1 ratio to group 1 or group 2 using numbered, sealed, and opaque envelopes. The envelopes were computer-generated by an employee who was not directly involved in the training, skills testing, and data collection.

#### Sample size calculation

Sample size calculation was based on a previous study from our group [31]. From this data, we estimated an expected difference (τ) for the primary outcome (OSATS) to be three points which would reflect a relevant difference. Three points represent 8.6% of the maximum reachable global OSATS score (35 reachable points) and 4.3% of the maximum reachable task specific OSATS score (70 reachable points) and 2.9% of the total OSATS score (105 reachable points). The square root of the measurement variance (σ_e_^2^) was set to 4.5. Thus, based on the sample size formula for the unpaired *t*-test with the proposed adjustment for the crossover design, a sample size of 36 would be required to have an 80% probability to detect the aforementioned difference [32, 33]. To account for potential drop-outs, 10% were added leading to a total sample size of 40 participants.

#### Statistical analysis

The statistical analysis including sample size calculation was done in cooperation with the Institute for Medical Biometry and Computer Science of the University of Heidelberg. Statistical analysis and descriptive statistics were then performed with the SPSS software (version 25.0, IBM SPSS Inc., Chicago, Illinois, USA) and data was given as absolute frequency and as mean ± standard deviation. The evaluation was carried out under consideration of the crossover design. Differences between the cholecystectomies were assessed using the *t*-Test for independent samples in case of parametric data and the Mann–Whitney U test for independent sample in case of non-parametric data. For binary endpoints group differences were calculated using the Chi-square test. Multivariable regression was performed to assess influence of demographical parameters and personal characteristics on surgical performance. A *p*-value of *p* < 0.05 was considered statistically significant.

## Results

### Demographics

In total, 40 medical students participated in the randomized crossover study (20 per group). 39 of them completed the entire study protocol between June and October 2019, one student from group 2 could not perform the RAC due to logistical issues. No differences were observed between groups for baseline characteristics (Table 3).

**Table 3:** Participants’ baseline characteristics stratified by group (*n* (%))

	Group 1 (*n* = 20)	Group 2 (*n* = 20)
Sex (male)	12 (60%)	10 (50%)
Age (years)	22.0 ± 1.0	22.0 ± 1.0
Medical school module of surgery completed	8 (40%)	10 (50%)
Dominant hand (right)	19 (95%)	14 (70%)
Video game activity (yes)	15 (75%)	11 (55%)
Sports activity (yes)	19 (95%)	18 (90%)
Playing a musical instrument (yes)	16 (80%)	15 (75%)
Use of E-learning platforms (yes)	15 (75%)	12 (60%)
Use of online teaching videos for surgery (yes)	9 (45%)	6 (30%)

### Primary endpoint

#### Objective structured assessment of technical skills OSATS score

**Fig. 4:**
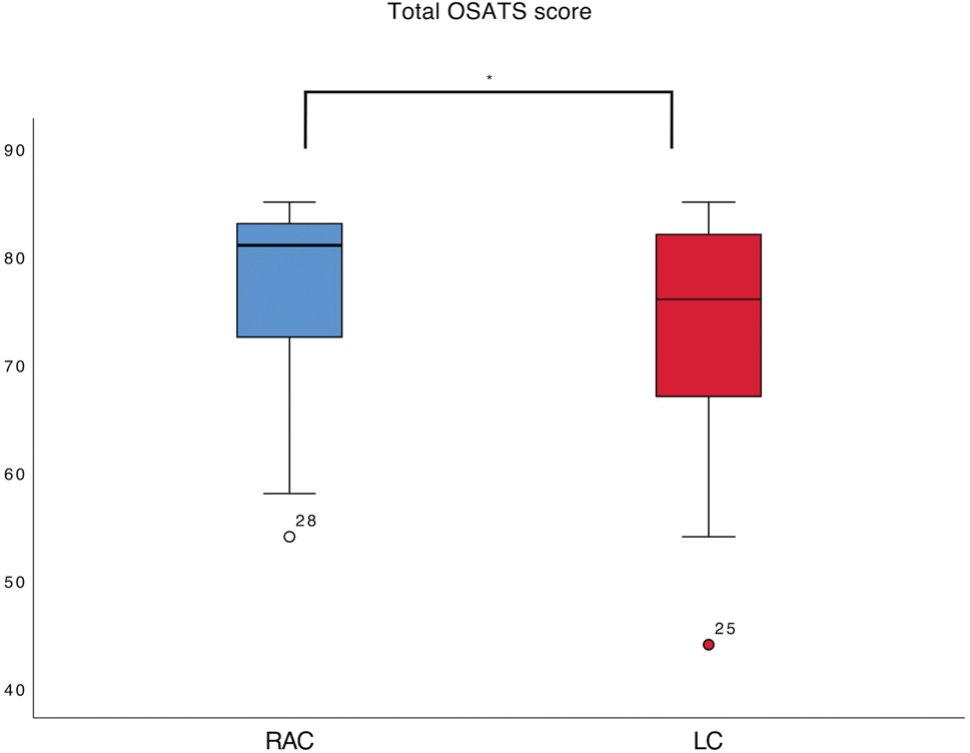
Total Objective Structured Assessment of Technical Skills (OSATS) score for robotic-assisted cholecystectomies (RAC) and laparoscopic cholecystectomies (LC), Mann–Whitney U, *significant for *p* < 0.05

The total OSATS score was higher for RAC than for LC (RAC = 77.4 ± 7.9 vs. LC = 73.8 ± 9.4; *p* = 0.025) OSATS scores evaluation shows that the students scored significantly higher during RAC for the global OSATS score (*p* = 0.012) and the task specific OSATS score (*p* = 0.037) than for LC (Fig. 4).

### Secondary endpoints

#### Operating time

**Fig. 5:**
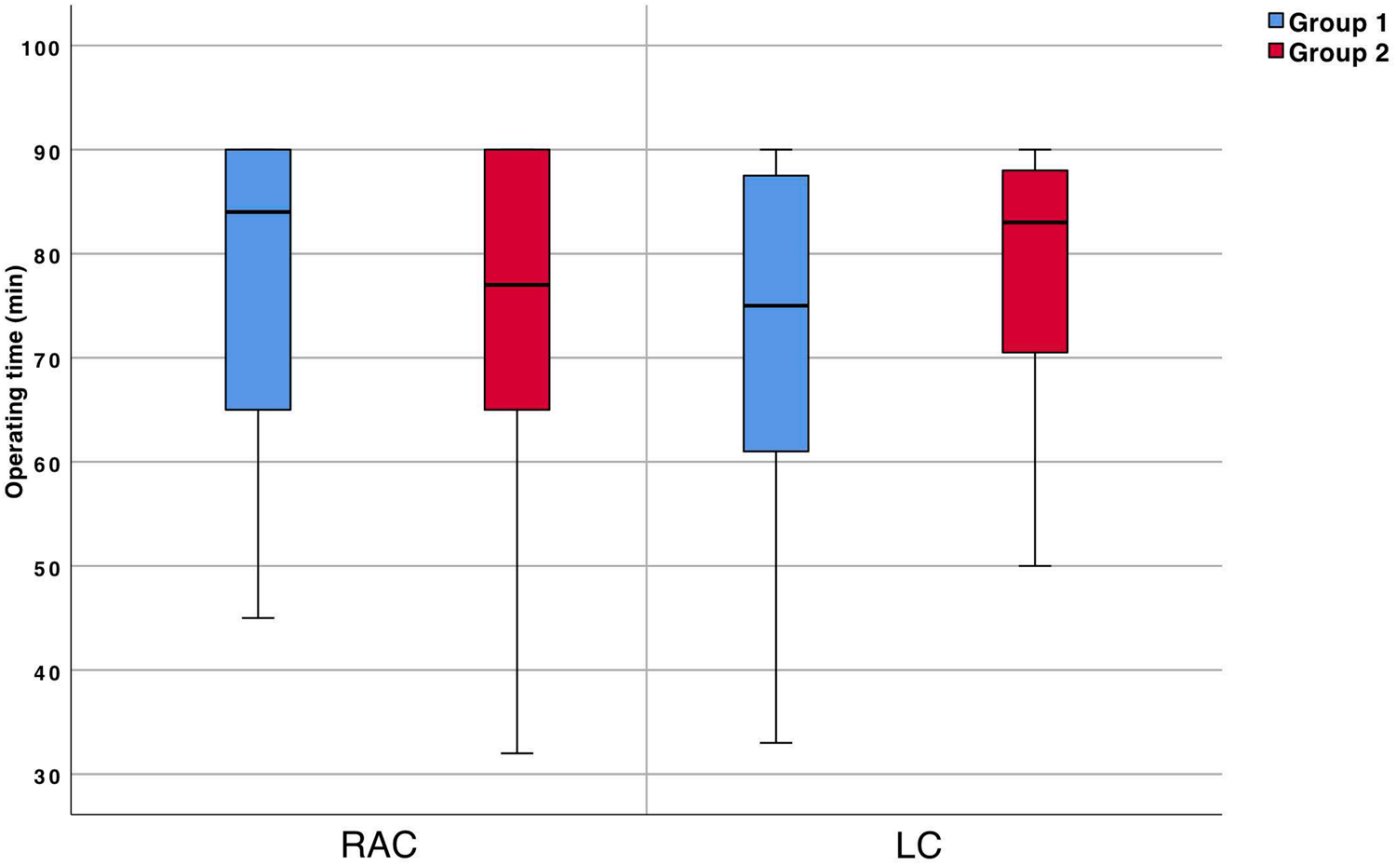
Operating time (min) for robotic-assisted cholecystectomies (RAC) and laparoscopic cholecystectomies (LC). Mann–Whitney U, *significant for *p* < 0.05

There were no significant differences between RAC and LC for operating time (RAC = 77.0 ± 15.3 vs. LC = 75.5 ± 15.3 min; *p* = 0.517) (Fig. 5).

### Difficulty of laparoscopic cholecystectomy

There was no difference in gallbladder size (surface of gallbladder bed in cm^2^) between all RACs and all LCs (RAC = 19.3 ± 4.1 vs. LC = 19.6 ± 3.9, *p* = 0.662). The difficulty of each cholecystectomy was assessed by the study director, using the visual analog scale (VAS) [34] (from 1 (very easy) to 10 (very difficult)) and showed no differences between all RACs and LCs (RAC = 4.3 ± 1.6 vs. LC = 4.3 ± 1.4; *p* = 0.932).

### Complications

While performing the RAC, the students injured the liver less compared to the LC. Even though less minor liver damage was recorded during the LC than during the RAC, the students caused more frequently major liver damages while performing LC. There were no differences for gallbladder perforations and lesions caused on the cystic artery and the cystic duct between RAC and LC. The students made less clipping mistakes with RAC than with LC. The combined endpoint showed a significant difference with less complications for RAC compared to LC. These results are resumed in Table 4.

**Table 4:** Complications for all robotic-assisted cholecystectomies (RAC) and laparoscopic cholecystectomies (LC)

	RAC (%) *n* = 39	LC (%) *n* = 40	*p*-value
Liver damage			
No damage	5 (12.8%)	1 (2.5%)	0.001*
Minor damage	28 (71.8%)	18 (45.0%)	
Major damage; numerous small lesions	6 (15.4%)	21 (52.5%)	
Gallbladder perforation			
No damage	26 (66.7%)	29 (72.5%)	0.626
Minor damage	10 (25.6%)	7 (17.5%)	
Major damage	3 (7.7%)	4 (10.0%)	
Damage on artery/duct			
No damage	31 (79.5%)	31 (77.5%)	0.670
Reparable damage	5 (12.8%)	5 (12.5%)	
Irreparable damage	3 (7.7%)	4 (10.0%)	
Clips placed			
Clips placed correctly	34 (87.2%)	29 (72.5%)	0.039*
Clipping slightly out of correct place	4 (10.3%)	6 (15.0%)	
Structural damaged, clips not closing	1 (2.5%)	5 (12.5%)	
Combined endpoint			
No major damage on liver, gallbladder, vessels or through clips	29 (74.4%)	14 (35.0%)	0.006*
Major damage on liver, gallbladder, vessels or through clips	10 (25.6%)	26 (65.0%)	

Mann–Whitney U. Number of participants (absolute or %)

*Significant for *p* < 0.05

A multivariable regression was performed to evaluate the influence of the baseline characteristics on surgical performance of the students (OSATS score, operating time, combined endpoint). Playing video games was a significant predictor on surgical performance measured with the OSATS scores. Students who played video games reached higher ranges for the task specific OSATS scores than students who did not play video games (*p* = 0.010). Also, students using online teaching videos for surgery in their spare time reached higher ranges for operating time than students who did not (*p* = 0.018). No further differences were seen in regard to the baseline characteristics and surgical performance.

### Subjective workload assessment

**Fig. 6:**
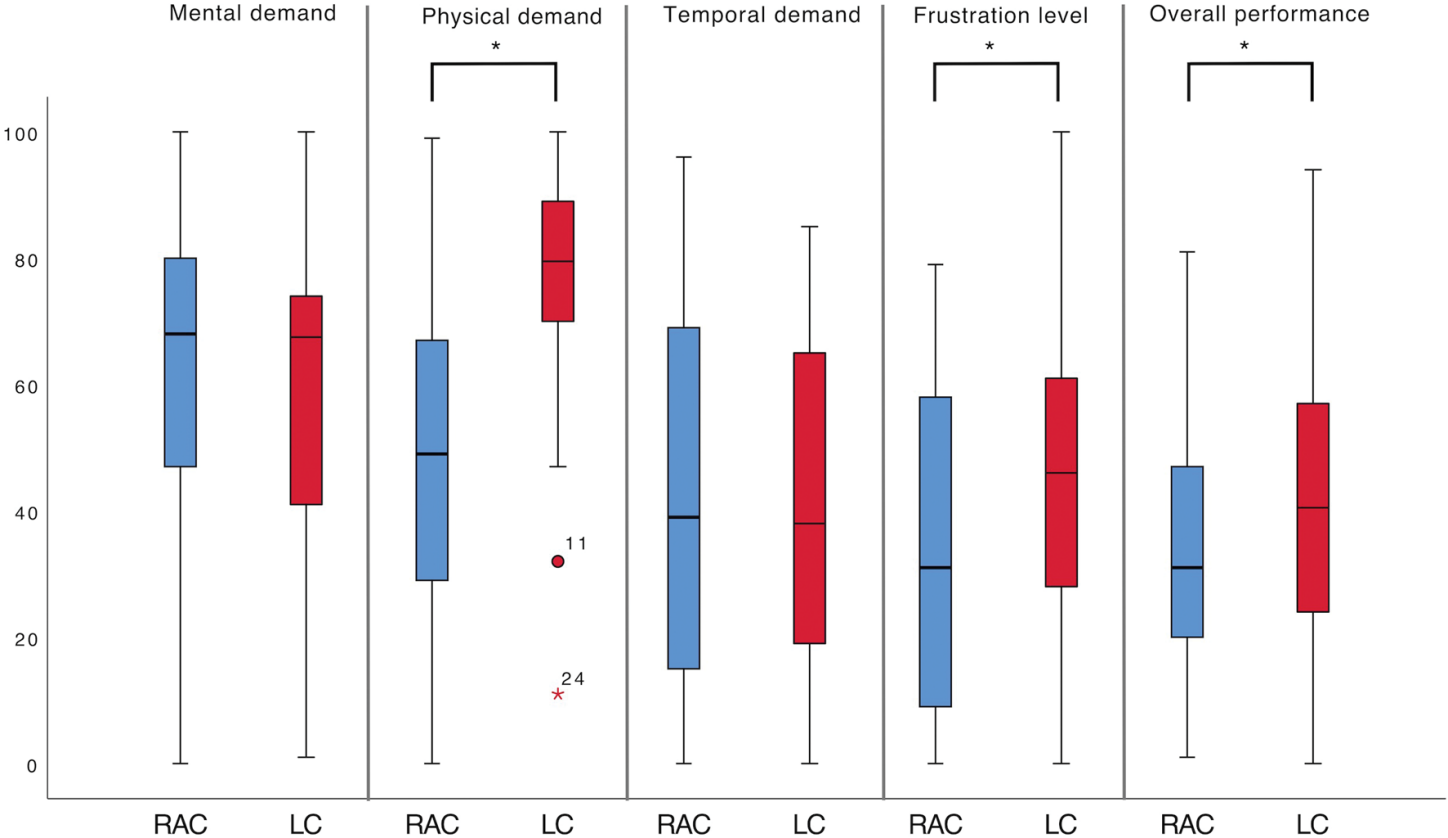
Subjective workload assessment (NASA TLX) for all robotic-assisted cholecystectomies (RAC) and laparoscopic cholecystectomies (LC). Mental, physical and temporal demand & frustration level: 0 (very low); 10 (very high). Overall performance: 0 (perfect); 100 (failure). Mann–Whitney U and independent t-Test, *significant for *p* < 0.05

The subjective workload assessment obtained using the NASA TLX questionnaire showed that students perceived the RAC significant less physically demanding than the LC (*p* < 0.001) and a lower frustration level (*p* = 0.023). The overall performance was perceived as better by the students after performing the RAC than the LC (*p* = 0.021) (Fig. 6).

The results for the ASQ showed that the students seemed more satisfied with the ease of completing the cholecystectomy on the robotic system than with conventional laparoscopy (RAC = 2.6 ± 1.5 vs. LC = 3.2 ± 1.4; *p* = 0.006). There was no difference for satisfaction with the amount of time needed to complete the task (RAC = 3.4 ± 2.1 vs. LC = 3.4 ± 2.0; *p* = 0.731) and the satisfaction of informational support (RAC = 2.3 ± 1.7 vs. LC = 2.2 ± 1.7; *p* = 0.895) (scale of 1 (I agree) to 7 (I do not agree)).

The self-made questionnaire assessing the physical workload and the ease to work on each operating system showed significant differences between the RACs and the LCs. Students perceived the performance on the robotic system and the instrument manipulation as less physical demanding, could find a more comfortable working position, could use the instruments in a more appropriate manner and were more eased to evaluate depths and distances than with CL. On the other hand, the students felt more isolated from the outside world while performing the RAC than during the LC (Table 5).

**Table 5:** Physical workload assessment and ease to perform cholecystectomy for all robotic-assisted cholecystectomies (RAC) and laparoscopic cholecystectomies (LC)

	RAC (Mean ± SD) *n* = 39	LC (Mean ± SD) *n* = 40	*p*-value
Comfortable working position	4.0 ± 1.0	2.7 ± 1.0	< 0.001*
Physically demanding	2.4 ± 1.1	3.7 ± 0.9	< 0.001*
Manipulation of instruments physically demanding	2.3 ± 1.1	4.0 ± 0.9	< 0.001*
Appropriate instrument manipulation	4.1 ± 0.7	3.8 ± 0.7	0.028*
Ease in evaluating depth and distances	4.3 ± 0.7	3.9 ± 0.9	0.010*
Feeling of being cut off from outside world	2.7 ± 1.3	2.6 ± 1.3	0.024*

Mann–Whitney U and independent t-Test

*Significant for *p* < 0.05. This questionnaire used a 5-point Likert scale (1: I don’t agree, 5: I totally agree)

### ForceSense tissue interaction assessment

A correlation using Pearson’s correlation was shown between the first recording of each cholecystectomy and all recordings for the same cholecystectomy for mean non-zero force (*r* = 0.81 *p* ≤ 0.001) and force volume (*r* = 0.68 *p* = 0.014). The force measurements performed during the whole cholecystectomies showed no significant differences between the robotic-assisted and the laparoscopic cholecystectomies for maximum force (N), maximum impulse (Ns), force volume (N^3^) and mean non-zero force (N) (Table 6).

**Table 6:** Maximum Force (N), maximum Impulse (Ns), Force volume (N^3^) and mean non-zero Force (N) for robotic-assisted cholecystectomies (RAC) and laparoscopic cholecystectomies (LC)

	RAC (Mean ± SD) *n* = 18	LC (Mean ± SD) *n* = 18	Difference (%)	*p*-value
Max. Force (N)	10.7 ± 6.0	9.2 ± 4.3	− 14.0	0.882
Max. Impulse (Ns)	987.5 ± 413.7	1185.9 ± 565.4	+ 20.1	0.588
Force volume (N^3^)	0.8 ± 0.6	1.0 ± 0.8	+ 25.0	0.349
Mean non-zero force (N)	1.8 ± 0.5	2.0 ± 0.7	+ 11.1	0.071

Mann–Whitney U

*Significant for *p* < 0.05

## Discussion

The present study compared RAS and CL in the initial learning phase for minimally invasive cholecystectomy in novices. The operative performance was significantly better in the OSATS score with RAC than with LC and there were less intraoperative complications for RAC than for LC. There was no difference for operating time. Workload assessments using the NASA TLX, the ASQ and a self-made questionnaire showed that students perceived lower frustration levels, felt less physical demand and were more satisfied with their performance with RAC than with LC. Force interaction assessment showed no difference in absolute maximum force, maximum impulse, force volume and mean non-zero force applied to the tissue during RAC and LC.

Participants achieved higher OSATS performance scores with RAC than with LC. RAS helped students to perform the procedure safer and more comfortably than with CL in their initial learning curve. Previous studies compared learning curves between RAS and CL and showed shorter learning curves for the robotic system for basic skills and for performing complex tasks such as laparoscopic suturing [35, 36]. This is in line with the results of the present study since the students performed relatively complex procedural tasks for surgical novices with higher scores and less complications with RAS than with CL. The operating times were not different between RAC and LC as opposed to some clinical trials that showed longer operating times for RAS than for CL [37]. In laparoscopic training, time is considered as an established and objective assessment parameter, and it enables the measurement of learning success to a limited extent [38]. Previous studies showed that RAS can lead to longer operating times and this generates a considerable cost increase in case of equal clinical outcomes and complications compared to CL [39–43]. The students in the present study had little prior experience in both RAS and CL except for a 10-h basic training for both entities. The current study can thus be seen as independent from the bias of prior experience which renders direct clinical comparisons often difficult even in randomized trials. Prior studies showed that RAS has a distinct a distinct learning curve and the docking times that are included in operative times often reflect team training rather than surgical performance [44, 45]. The present study thus showed possible advantages of RAS over CL in the early learning phase when little prior but equal experience for both modalities exists.

The subjective workload assessment obtained using the NASA TLX questionnaire showed that the perception of physical demand, frustration level and overall performance were significantly lower with RAC than with LC. Students performed RAS with lower stress levels than with CL, they felt more confident with their performance and the cholecystectomy was perceived as less physical demanding. Similar results were shown previously while comparing physical and cognitive ergonomic workloads between RAS and CL for basic skill training [46]. The results for the ASQ showed that the students were more satisfied with the ease of completing the cholecystectomy with RAS than CL. No differences were seen for satisfaction with the amount of time needed to complete the task and the satisfaction of informational support as they were comparable between the groups. In addition, students perceived the performance of RAS and the instrument manipulation as less physical demanding, could find a more comfortable working position, could use the instruments in a more appropriate manner and were more eased to evaluate depths and distances than with CL. On the other hand, students felt more isolated from the outside world while performing the RAC than the LC. It is known from other studies that increased workload and higher frustration and stress levels can lead to inferior task performance and increase the probability of doing mistakes during a procedure [47]. This is in line with the results from the present study that showed lower workload and less frustration with RAC than with LC and at the same time the operative performance was better, and the complications were less with RAC than with LC. The robotic system seems to have enabled students to perform the cholecystectomy in a mentally more favorable way than CL.

For the force measurement, a correlation could be shown between the first recording of each cholecystectomy and all recordings for the same cholecystectomies. Therefore, the force measurements are applicable to the entire procedures. The force measurements showed that the maximum absolute force applied during the procedure was slightly higher during the RACs as the LCs, but the mean non-zero force, maximum impulse and force volume was higher during the LCs. These minimal differences showed that the lack of haptic feedback on the robot did not influence the student’s performances even while performing a cholecystectomy for the first directly on the robotic system. Haptic feedback is already reduced in CL, but it is currently non-existent in most commercially available RAS systems. The role of haptic feedback plays an important role in CL as it enables the surgeon to “feel” the tissue and perform in a safer way. For RAS surgeons have to learn visual haptics to compensate for this lack of tissue feeling. The value of visual haptics (and also auditory feedback) to help perform safer and better in the surgical setting was demonstrated in various studies [48, 49]. The acquisition of visual haptics by surgeons is seen as a complex learning task, but it has also been demonstrated that beginners could quickly experience the perception of haptic feedback via visual clues. Therefore, the learning of visual haptics which is necessary to perform RAS does not seem to compare unfavorable to the difficulties in the initial learning curve for CL, such as 2D-3D coordination, Pivot- and Fulcrum effects, and restricted degrees of freedom [50]. This could be a further explanation why RAS has shown advantages over CL in the early learning curve of cholecystectomy in the present study. The force measurement could successfully be implemented into a training for both CL and RAS, so applied forces can be shown. This may be used as feedback for trainees in further studies in this domain.

The generalizability and transferability of the present study to the operating room needs to be proven. The participants were medical students with 10 h of prior training in MIS and the cholecystectomies were performed on cadaveric porcine model. Previous studies evaluated the effect of robotic training to improve surgical skills between experts and novices. The results showed that using robotics helped novices to improve their surgical performance in a more evident way than for experts. However, the robotic system was still useful for improving the expert’s economy of motion [51, 52]^.^ It should be mentioned that RAS and CL require distinct skills and that there is limited transfer of basic skills between CL and RAS basic techniques [53–55]. Robotic training may allow less experienced minimally invasive surgeons who are proficient in open surgery to perform more complex MIS procedures without having to develop advanced CL skills first. Nevertheless, before acquiring advanced skills in laparoscopy or robotics, the acquisition of basic and fundamental MIS skills is necessary [56, 57]. Cholecystectomy often represents one of the first MIS procedures performed by surgeons in training, and therefore the initial learning curve is especially important. LC can be trained outside of the operating room in a safe and realistic way using box trainers and pulsatile organ perfusion trainers [58]. Previous training in box trainers and virtual reality trainers showed improvement of surgical skills in regard to OSATS scores and time while performing a cadaveric LC compared to no training in various studies with different training curricula [59, 60]. After simulation training there is a proven transition to the real operating room with supervised procedures in surgical training curricula to improve the early learning curve [61]. Proficiency-based preclinical training has shown to impact the initial learning curve for LC in a positive way [62].Various training modalities have been developed to enable an optimized and safe training of LC as well as further laparoscopic procedures and skills [63, 64]. Integrating robotic surgery in laparoscopic training could enable the implementation of multimodal training curricula which have shown to help novices to improve basic skills and overcome the initial learning curve in laparoscopy. Junior and senior resident’s surgical skill level and operative performance can be improved, and operative time reduced thanks to a multimodal training structures [65]. Surgical robotic training might expand even more in the next years as new robotic systems are being developed and are enrolled on the market [66]. Including RAS to MIS training curricula could allow young surgeons and novices to improve their minimally invasive surgical skills and learn to perform procedures safer for the entire spectrum of minimally invasive surgery. Including MIS and RAS training with assessment of skills and learning curves in student education also bears the potential of a positive selection of surgical residents and could aid in finding and attracting suitable candidates for residency programs based on their “natural” skills.

As has been shown in other studies, the surgical performance of novices was positively influenced by video gaming activity and using online teaching videos for surgery in the present study while there were no gender differences [67–69].

## Limitations

A limitation of the present study is the fact, that the participants only performed two cholecystectomies each and therefore it can only be reasoned about the initial learning curve. The question of the present study was specifically the initial performance of cholecystectomy. However, we agree that it would certainly be desirable to perform another study with more longitudinal learning curve comparison of RAC and LC. This was not feasible in the setting of the current study due to time and resource restrictions and would have meant to have the students perform at least 10 cholecystectomies each which would have been beyond the scope of their mandatory courses and the setting of the present study. Further studies should compare the learning curve between robotics and laparoscopy on a larger scale and let the participants perform more cholecystectomies. Furthermore, financial and logistical difficulties might limit the extension of the presented training program to different settings since MIS training facilities especially with robotic systems are not available everywhere. The present study showed, however, that performing a full procedure on the robotic system was feasible for laparoscopic novices and that they could exploit the advantages of this system to perform better compared to CL. Also, the cholecystectomies were performed by laparoscopic novices on cadaveric porcine livers placed in a box trainer, this has, however, been shown to have high face validity [70]. It has been shown that training on porcine models in a training center resulted in a better performance in the operating room in comparison to non-trained study participants [71, 72],. So, training in the setting presented in this study could allow the transfer of skills to the operating room. The study was planned with a relatively small difference of three points in OSATS score considered as relevant difference for the sample size calculation. This represents 2.9% of the maximum reachable score and 3.9% of the actual mean score of the RAC group. There have recently been studies that could show the importance of surgeon’s technical skill as assessed by their peers with OSATS or similar skill assessment methods. These studies found considerable improvements both in complication rates but also in operative time and even in oncological outcome with better skill scores [73–75]. It thus seems reasonable to perform studies with surgical performance as primary endpoint although there has not been a consensus on the minimum relevant difference in OSATS scores that can be translated into clinically relevant benefits so far. This question should be addressed in future studies. The force assessment with ForceSense was a pilot evaluation based on force interaction assessment during RAC and LC. The low sample size for this pilot evaluation and the fact that recordings were limited in time are limitations to this evaluation. The pilot study showed great potential for the use of force assessment in this setting with the cadaveric porcine model and there were no differences between RAC and LC. There is, however, high potential for use of force feedback that can be derived from this system as seen by the complications that happened during the training procedures in the present study. This will be evaluated as a feedback mechanism to enhance training in future studies as well as automated methods of skill assessment to facilitate performance assessment and feedback [76, 77].

## Conclusions

In the present study, RAC was superior to LC in the early learning phase as reflected by better operative performance in OSATS score and less overall complications while the operating times were similar. The subjective workload assessment showed that students perceived less physical workload, reached lower frustration, were more satisfied with their overall performance, and were more satisfied with the ease of completing the task with RAC than with LC. There was no difference in force applied to the tissue between RAC and LC. RAC seems thus to have advantages over LC in the early leaning phase for beginners in minimally invasive surgery.

## Abbreviations

LC: Laparoscopic cholecystectomy
RAC: Robotic-assisted cholecystectomy
OSATS: Objective structured assessment of technical skill
NASA TLX: NASA task load index
ASQ: After-scenario-questionnaire
MIS: Minimally invasive surgery
RAS: Robotic-assisted surgery
CL: Conventional laparoscopy
